# Laparoscopic management of bowel perforation secondary to levonorgestrel-releasing intrauterine device migration: a case report and review of literature

**DOI:** 10.1093/jscr/rjae522

**Published:** 2024-08-30

**Authors:** Jeong Hee Han, Eun Hee Yu, Jong Kil Joo, Min Ju Kim, Jung Bum Choi, Hyuk Jae Jung, Hong Jae Jo, Byoung Chul Lee

**Affiliations:** Department of Surgery, Pusan National University School of Medicine, Pusan National University Hospital Biomedical Research Institute, Busan 46241, Republic of Korea; Department of Obstetrics and Gynecology, Pusan National University School of Medicine, Pusan National University Hospital Biomedical Research Institute, Busan 46241, Republic of Korea; Department of Obstetrics and Gynecology, Pusan National University School of Medicine, Pusan National University Hospital Biomedical Research Institute, Busan 46241, Republic of Korea; Department of Surgery, Pusan National University School of Medicine, Pusan National University Hospital Biomedical Research Institute, Busan 46241, Republic of Korea; Department of Surgery, Pusan National University School of Medicine, Pusan National University Hospital Biomedical Research Institute, Busan 46241, Republic of Korea; Department of Surgery, Pusan National University School of Medicine, Pusan National University Hospital Biomedical Research Institute, Busan 46241, Republic of Korea; Department of Surgery, Pusan National University School of Medicine, Pusan National University Hospital Biomedical Research Institute, Busan 46241, Republic of Korea; Department of Surgery, Pusan National University School of Medicine, Pusan National University Hospital Biomedical Research Institute, Busan 46241, Republic of Korea

**Keywords:** levonorgestrel-releasing intrauterine device (LNG-IUD), intrauterine device (IUD), bowel perforation, IUD migration

## Abstract

Bowel perforation secondary to a levonorgestrel-releasing intrauterine device is exceptionally rare. We present the case of a woman who exhibited abnormal findings during a colonoscopy examination. Despite undergoing an intrauterine device (IUD) insertion procedure for contraception in 2000, attempts for its removal in 2007 were unsuccessful due to the inability to locate the IUD. In 2022, she presented with intermittent hematochezia and lower left abdominal pain. Subsequent colonoscopy and abdominal computed tomography confirmed the presence of the IUD penetrating the uterine wall and entering the colon. Laparoscopic anterior resection was performed, and the patient’s postoperative recovery was uneventful, indicating the viability of laparoscopic treatment as a valuable option.

## Introduction

Since its introduction in the late 1990s, the levonorgestrel-releasing intrauterine device (LNG-IUD) has emerged as a widely utilized contraceptive method among millions of women globally [1]. Furthermore, it is increasingly prescribed for its therapeutic benefits in addressing conditions such as menorrhagia, dysmenorrhea, and endometriosis [2, 3]. While uterine perforation secondary to an IUD is a rare occurrence, bowel perforation resulting from IUD migration is an even rarer and more serious complication [4]. Notable, most documented cases of bowel perforation related to IUD involve copper IUD [5, 6]. Here, we report an unusual case of bowel perforation due to migration of an LNG-IUD.

## Case report

**Figure 1:**
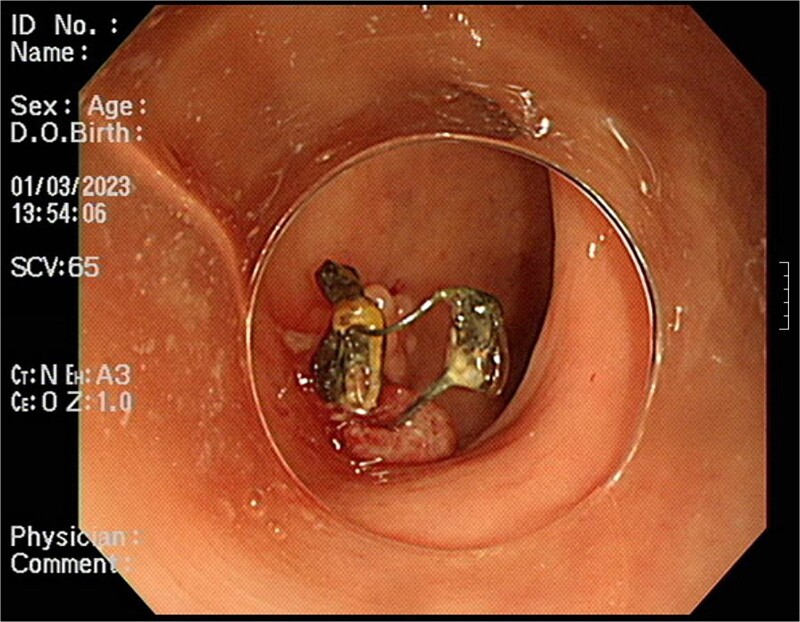
Foreign body penetrating the sigmoid wall with surrounding granulation tissue observed during colonoscopy.

**Figure 2:**
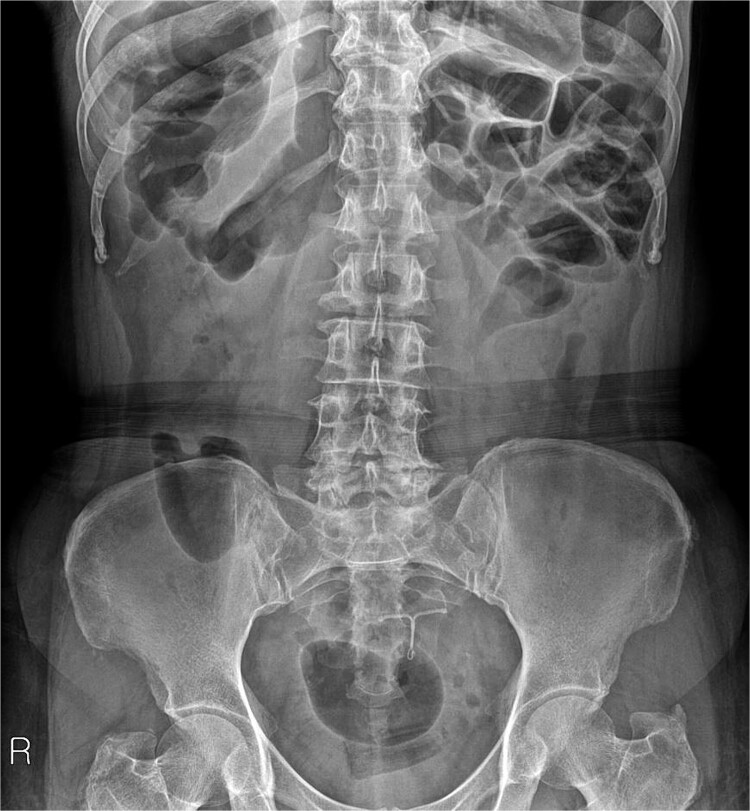
IUD identified on abdominal simple X-ray.

**Figure 3:**
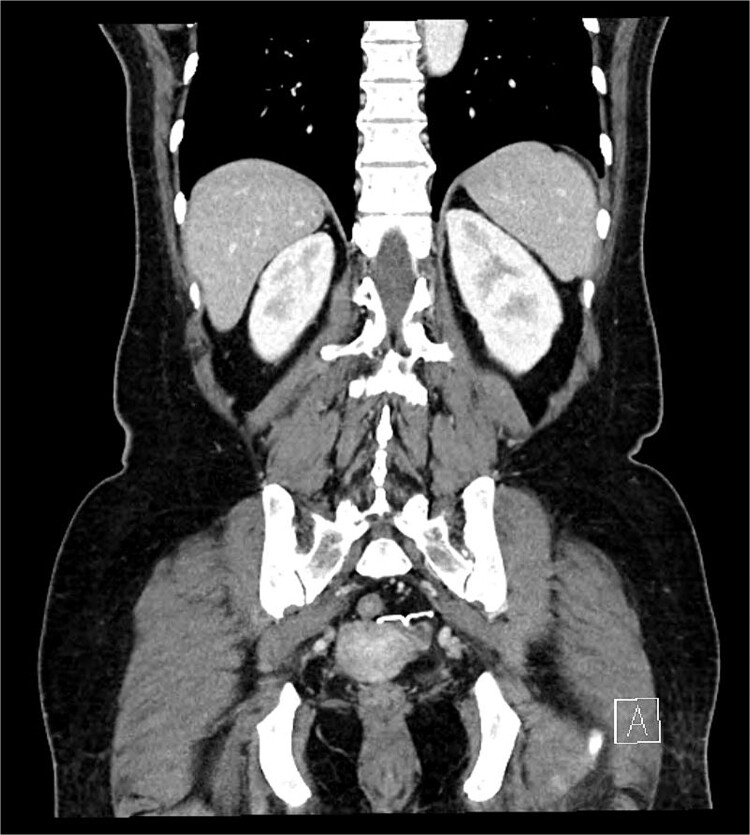
IUD positioning adjacent to the left uterus and extending into the colon, observed on CT.

A 54-year-old woman, gravida 4, para 2, presented at the outpatient department with abnormal colonoscopy findings. She had a history of hypertension and underwent an IUD insertion for contraception one year after delivery in 2000. However, in 2007, when she visited the clinic for its removal, the IUD was not found on pelvic examination and ultrasound. The patient assumed that the IUD had been expelled spontaneously. Six months before her 2022 hospital visit, she experienced intermittent rectal bleeding and lower left abdominal pain, leading her to seek medical attention. Her medical history and physical examination, including a digital rectal examination, showed no notable abnormalities except for mild left lower quadrant tenderness. Routine laboratory investigations yielded unremarkable results. During colonoscopy, a foreign body was observed penetrating the sigmoid wall, surrounded by granulation tissue (Fig. 1). Subsequently, an abdominal simple X-ray and computed tomography scan were performed, revealing that the foreign body was an IUD, located next to the left side of uterus and entering the sigmoid colon (Figs 2 and 3).

**Figure 4:**
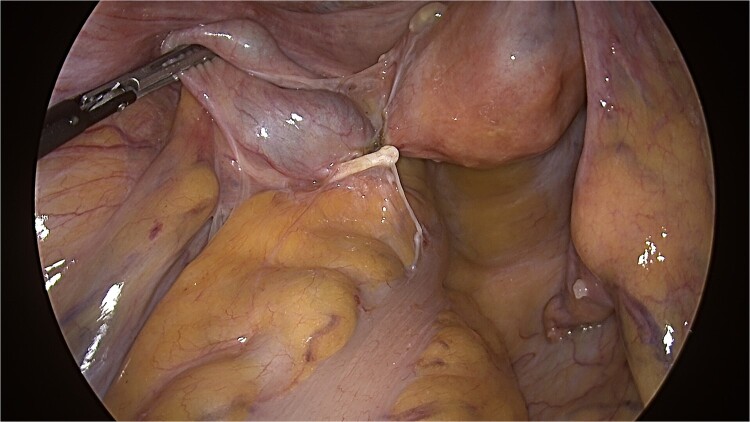
IUD with firm adhesions between the left adnexa and rectum observed during laparoscopy.

The patient underwent laparoscopic anterior resection with salpingo-oophorectomy due to tight adhesions formed by the LNG-IUD, which had firmly adhered between the left adnexa and rectum (Fig. 4). The patient’s postoperative course was discharged without any complications.

## Discussion

IUDs are regarded as one of the most effective, safe, cost-effective, and long-acting reversible methods of contraception [7]. However, while uterine perforation associated with LNG-IUDs has been reported with an estimated frequency of approximately 8.5%, cases of LNG-IUD-induced bowel perforation are exceedingly rare [7]. The mechanisms of perforation include immediate traumatic perforation occurring during the procedure and delayed ‘secondary’ perforation due to gradual erosion through the uterine wall. Risk factors for perforation include insertion by less experienced clinicians, lactation, postpartum insertion, lower parity, and a higher number of previous abortions. However, the associations are either weak or inconsistent, so causality has not been demonstrated [8, 9].

The manifestation of IUD migration varies according to the migration site and type of IUD. In cases of bowel perforation caused by an IUD, the majority of patients experienced abdominal pain, but were otherwise in good health. Although abdominal pain could indicate bowel perforation, such perforation may not always present symptoms. In asymptomatic cases, migrated IUDs might go unnoticed for extended periods of time [4] . Also, the majority of documented instances of bowel and urinary bladder perforation were asymptomatic upon diagnosis [10].

The controversy surrounding surgical intervention for symptomatic uterine perforation extends to the management of an asymptomatic dislocated IUD within the abdomen, which remains contentious. Some researchers suggested that adhesions following uterine perforation induced by the IUD typically occur nearby, minimizing the likelihood of intestinal obstruction and thus questioning the necessity for removal of the perforated IUD [11, 12]. However, the World Health Organization recommends the removal of all displaced IUDs within the abdomen following uterine perforation [13]. In addition, it is prudent to consider that conservative management may pose significant risks, especially for symptomatic women, given the documented instances of bowel obstruction and perforation associated with IUDs that have penetrated the uterus [14, 15]. Moreover, considering the potential to alleviate pain or bleeding in symptomatic cases, and given the high success rate of laparoscopic retrieval alongside continuous advancements in minimally invasive surgical techniques, surgical treatment remains optimal [4].

In cases of intestinal perforation caused by an IUD, removal options include endoscopic, laparoscopic, or open surgical approaches. Colonoscopy is recommended for IUD removal, especially when the device is embedded within the colon wall. However, if intestinal perforation is incidentally discovered during endoscopy, it’s advisable to consider removal at a surgical facility capable of managing potential complications rather than immediate extraction. Unsuccessful attempts at removal via colonoscopy may necessitate subsequent surgical intervention due to the risks of perforation and bleeding [15]. Surgical removal can be accomplished through either laparoscopy or open laparotomy, with laparoscopic techniques generally preferred for their minimally invasive nature, unless the complexity of the case requires open surgery.

In our case, the problem began when the possibility of displacement was not considered despite the IUD not being confirmed in its proper position when the patient visited for IUD removal. The onset of symptoms 15 years later indirectly suggests the potential for ongoing LNG-IUD migration even after displacement, highlighting the risk of bowel perforation not only with Copper IUDs but also with LNG-IUDs. Despite encountering severe adhesions involving the sigmoid colon and left adnexa, laparoscopic surgery was performed safely.

In conclusion, although rare, LNG-IUD poses a risk of bowel perforation through migration. If the IUD is located within the body but cannot be visualized within the uterine cavity via ultrasound, further investigation with a CT abdomen may be warranted. Depending on the location, procedures such as colonoscopy could also be considered. With recent advancements in laparoscopic and minimally invasive surgery, reluctance and risks toward surgical intervention have diminished compared to the past. Therefore, besides conservative management, surgical treatment may be considered as a valuable option.

## References

[1] French R , Van Vliet H, Cowan F, et al. Hormonally impregnated intrauterine systems (IUSs) versus other forms of reversible contraceptives as effective methods of preventing pregnancy. Cochrane Database Syst Rev 2004;2004:CD001776. 15266453 10.1002/14651858.CD001776.pub2 PMC8407482

[2] Abou-Setta AM , Houston B, Al-Inany HG, et al. Levonorgestrel-releasing intrauterine device (LNG-IUD) for symptomatic endometriosis following surgery. Cochrane Database Syst Rev 2013;1:CD005072. 10.1002/14651858.CD005072.pub3. 23440798

[3] Lethaby A , Hussain M, Rishworth JR, et al. Progesterone or progestogen-releasing intrauterine systems for heavy menstrual bleeding. Cochrane Database Syst Rev 2015;30:CD002126. 10.1002/14651858.CD002126.pub3. 25924648

[4] Gill RS , Mok D, Hudson M, et al. Laparoscopic removal of an intra-abdominal intrauterine device: case and systematic review. Contraception 2012;85:15–8. 10.1016/j.contraception.2011.04.015. 22067801

[5] Kho KA , Chamsy DJ. Perforated intraperitoneal intrauterine contraceptive devices: diagnosis, management, and clinical outcomes. J Minim Invasive Gynecol 2014;21:596–601. 10.1016/j.jmig.2013.12.123. 24462588 PMC6661232

[6] Reynolds-Wright JJ , Heller RL. Delayed presentation of uterine and bowel perforation following insertion of an intrauterine device. BMJ Sex Reprod Health 2019;45:224–5. 10.1136/bmjsrh-2018-200288. 31028168

[7] Van Grootheest K . Uterine perforation with the Levonorgestrel-releasing intrauterine device. Drug Saf 2011;34:83–8. 10.2165/11585050-000000000-00000. 21142273

[8] Reed SD , Zhou X, Ichikawa L, et al. Intrauterine device-related uterine perforation incidence and risk (APEX-IUD): a large multisite cohort study. Lancet 2022;399:2103–12. 10.1016/S0140-6736(22)00015-0. 35658995

[9] Rowlands S , Oloto E, Horwell DH. Intrauterine devices and risk of uterine perforation: current perspectives. Open Access J Contracept 2016;7:19–32. 10.2147/OAJC.S85546. 29386934 PMC5683155

[10] Aggarwal S , Jindal RP, Deep A. Intravesical migration of intrauterine contraceptive devices with stone formation. J Family Med Prim Care 2014;3:449–51. 10.4103/2249-4863.148147. 25657964 PMC4311363

[11] Adoni A , Chetrit AB. The management of intrauterine devices following uterine perforation. Contraception 1991;43:77–81. 10.1016/0010-7824(91)90128-3. 1825971

[12] Markovitch O , Klein Z, Gidoni Y, et al. Extrauterine mislocated IUD: is surgical removal mandatory? Contraception 2002;66:105–8. 10.1016/S0010-7824(02)00327-X. 12204783

[13] Group, R.o.a.W.S . Mechanism of action, safety and efficacy of intrauterine devices. World Health Organ Tech Rep Ser 1987;753:1–91. 3118580

[14] Loveless A , Dhari A, Kilpatrick CC. Perforated levonorgestrel-releasing intrauterine system resulting in small bowel obstruction. J Reprod Med 2014;59:611–3. 25552138

[15] Lee J , Oh JH, Kim J, et al. Incomplete removal of an intrauterine device perforating the sigmoid colon. Korean J Gastroenterol 2021;78:48–52. 10.4166/kjg.2021.041. 34312357 PMC12286488

